# Ubiquitin Ligase RNF146 Regulates Tankyrase and Axin to Promote Wnt Signaling

**DOI:** 10.1371/journal.pone.0022595

**Published:** 2011-07-25

**Authors:** Marinella G. Callow, Hoanh Tran, Lilian Phu, Ted Lau, James Lee, Wendy N. Sandoval, Peter S. Liu, Sheila Bheddah, Janet Tao, Jennie R. Lill, Jo-Anne Hongo, David Davis, Donald S. Kirkpatrick, Paul Polakis, Mike Costa

**Affiliations:** 1 Department of Cancer Targets, Genentech Research and Early Development, South San Francisco, California, United States of America; 2 Department of Protein Chemistry, Genentech Research and Early Development, South San Francisco, California, United States of America; 3 Department of Discovery Oncology, Genentech Research and Early Development, South San Francisco, California, United States of America; 4 Department of Research Pathology, Genentech Research and Early Development, South San Francisco, California, United States of America; 5 Department of Antibody Engineering, Genentech Research and Early Development, South San Francisco, California, United States of America; University of Birmingham, United Kingdom

## Abstract

Canonical Wnt signaling is controlled intracellularly by the level of β-catenin protein, which is dependent on Axin scaffolding of a complex that phosphorylates β-catenin to target it for ubiquitylation and proteasomal degradation. This function of Axin is counteracted through relocalization of Axin protein to the Wnt receptor complex to allow for ligand-activated Wnt signaling. AXIN1 and AXIN2 protein levels are regulated by tankyrase-mediated poly(ADP-ribosyl)ation (PARsylation), which destabilizes Axin and promotes signaling. Mechanistically, how tankyrase limits Axin protein accumulation, and how tankyrase levels and activity are regulated for this function, are currently under investigation. By RNAi screening, we identified the RNF146 RING-type ubiquitin E3 ligase as a positive regulator of Wnt signaling that operates with tankyrase to maintain low steady-state levels of Axin proteins. RNF146 also destabilizes tankyrases TNKS1 and TNKS2 proteins and, in a reciprocal relationship, tankyrase activity reduces RNF146 protein levels. We show that RNF146, tankyrase, and Axin form a protein complex, and that RNF146 mediates ubiquitylation of all three proteins to target them for proteasomal degradation. RNF146 is a cytoplasmic protein that also prevents tankyrase protein aggregation at a centrosomal location. Tankyrase auto-PARsylation and PARsylation of Axin is known to lead to proteasome-mediated degradation of these proteins, and we demonstrate that, through ubiquitylation, RNF146 mediates this process to regulate Wnt signaling.

## Introduction

Wnt signaling is a fundamental morphogenetic pathway of metazoans that is deployed in diverse settings throughout development to regulate processes such as cell fate specification, stem cell regeneration, and neuronal migration [1]. Wnt signaling can become deregulated through multiple mechanisms to produce cancer or other diseases, particularly colorectal cancer for which APC or β-catenin is mutated in approximately 95% of tumors [2]. Consequently, many mechanisms have evolved to control the level, activity, and subcellular localization of multiple Wnt pathway components [3]. For example, Wnt ligands and their access to receptors of the FZD family and coreceptors LRP5 and LRP6 are modulated by decoy receptors, such as SFRP1, and by heparan sulfate proteoglycans, such as glypicans. Intracellularly, the best characterized mode of Wnt signaling regulation is the degradation of β-catenin by a protein complex that includes Axin and APC. This complex mediates the phosphorylation of β-catenin by CK1 and GSK3, which then signals β-catenin ubiquitylation by the SCF^β-TrCP^ complex to target β-catenin to the proteasome for proteolysis.

Axin protein, present in two isoforms, appears to be the most quantitatively limiting component of the β-catenin degradation complex [4], [5]. When Wnts engage their receptors, LRP5/6 is phosphorylated and recruits Axin into the receptor complex at the plasma membrane, where GSK3 bound to Axin becomes inactivated, thus preventing β-catenin degradation [6]. The critical role of Axin in controlling β-catenin levels and Wnt signaling is reflected in the multiple mechanisms of regulating Axin protein abundance in cells. AXIN2 is a direct transcriptional target of TCF/LEF transcription factors, thus generating a negative feedback loop whereby Wnt signaling increases AXIN2 mRNA, and consequently protein, levels to ultimately downregulate β-catenin [7]. In contrast, AXIN1 is part of a positive feedback mechanism for Wnt signaling since signaling destabilizes AXIN1 protein [8]. In this mechanism, since AXIN1 phosphorylation by GSK3 normally stabilizes AXIN1 protein, Wnt-induced GSK3 inactivation destabilizes AXIN1. More recently, the poly(ADP-ribose) polymerase (PARP) tankyrase was shown to poly(ADP-ribosyl)ate (PARsylate) AXIN1 and AXIN2 proteins to mediate their proteasomal degradation [9]. Small-molecule inhibitors of tankyrases TNKS1 and TNKS2 can downregulate Wnt signaling, and they also block the accumulation of ubiquitylated Axin upon proteasome inhibition. The ubiquitin E3 ligase SMURF2 also has been reported to ubiquitylate and degrade Axin [10].

Ubiquitylation is a fundamental mechanism for regulating the stability, interaction, and subcellular localization of many proteins, thereby controlling the activity of signaling pathways [11]. Ubiquitin molecules in polyubiquitin chains can be linked to each other through any one of seven Lys residues (or through the N-terminus), and this linkage type, or mixture of linkage types, can specify the fate of the attached protein. K48- or K11-linked polyubiquitin predominantly targets proteins for degradation by the 26S proteasome, whereas K63 linkage typically mediates protein-protein interactions or targets proteins for lysosomal degradation. Ubiquitin E3 ligases confer the substrate specificity of ubiquitylation by binding both the substrate and an ubiquitin-conjugating E2 enzyme, facilitating the transfer of ubiquitin from E2 to substrate. There are more than 600 human E3 ligases, and the largest structural class contains a RING domain that binds an E2. Together, the E2-E3 pair specifies the ubiquitin linkage type synthesized [12].

PARsylation can also control the function and localization of some proteins, and there are 17 human PARP family members, including TNKS1 and TNKS2 [13]. For example, tankyrase PARsylates TRF1 protein to inhibit its binding to telomeres, and this allows telomerase to access and lengthen telomeres [14]. Tankyrase-mediated PARslyation may sometimes be linked to ubiquitylation and protein degradation, as mentioned above for the apparent role of tankyrase in promoting the ubiquitylation and proteasome targeting of Axin [9]. Release of PARsylated TRF1 from telomeres results in TRF1 degradation by the proteasome [15]. Also, inhibition of tankyrase activity with a small-molecule inhibitor suggests that auto-PARsylation can translocate tankyrase from epithelial cell lateral membranes into the cytoplasm for ubiquitylation and proteasomal degradation [16].

In this report, we identify the E3 ligase RNF146 as a positive regulator of Wnt signaling that ubiquitylates and destabilizes Axin and tankyrase. We also show that tankyrase PARsylation activity reciprocally destabilizes RNF146 protein. Both RNF146 and auto-PARsylation activity also prevent tankyrase aggregation at a centrosomal location. Our results suggest that RNF146, tankyrase, and Axin form a complex in which ubiquitylation and PARsylation of all three proteins mediate their proteasomal degradation.

## Results

### RNF146 is a Positive Regulator of Wnt Signaling

**Figure 1:**
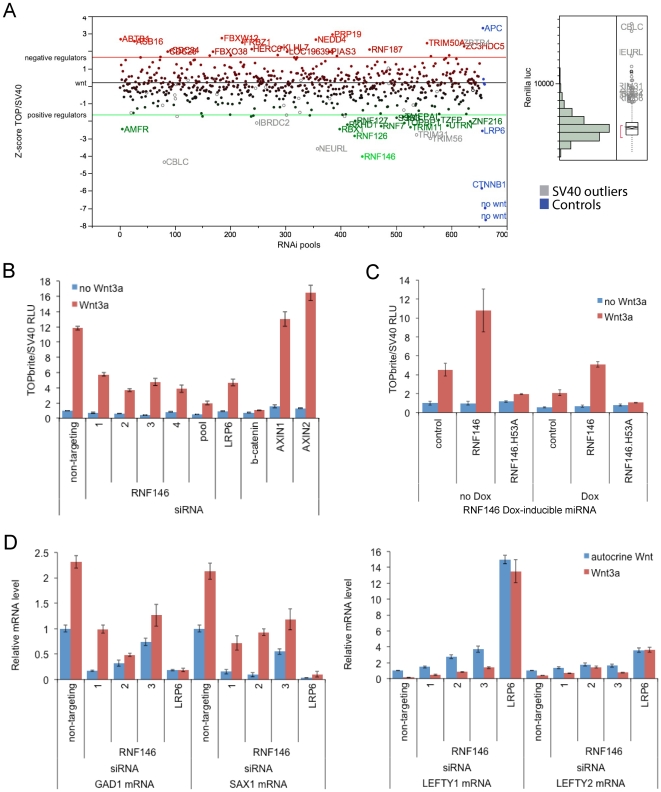
RNF146 positively regulates Wnt signaling. (A) Scatter plot of Z-scores for the RNAi screen of human E3 ligase siRNA pools in Wnt3a-stimulated HEK293 cells stably expressing dual luciferase reporters [17]. Z-scores on the *y* axis represent TOPbrite Wnt reporter values normalized to SV40 reporter values. Positively and negatively regulating siRNA pools that deviate from the Z-score cut-off lines are shown in green and red, respectively, by gene name. Control siRNAs targeting CTNNB1 (β-catenin), LRP6 and APC, as well as control wells not induced with Wnt3a protein, are depicted in blue but were excluded from the distribution analysis. SV40 reporter values are plotted to the right, with outliers deviating from the normal distribution listed in gray on both graphs. (B) RNF146 RNAi by transient transfection of four individual and pooled siRNAs in HEK293 cells showing inhibition of the Wnt3a response (red) and lack of nonspecific effects on uninduced (blue) reporter activity. Inhibition by LRP6 and β-catenin siRNAs, and activation by AXIN1 and AXIN2 siRNAs, are shown as controls. Error bars in this and all figures represent the standard deviation of at least three replicate samples. (C) RNF146 RNAi in HEK293T cells stably expressing doxycycline (Dox)-inducible miRNA targeting RNF146, and transiently transfected with TOPbrite Wnt luciferase reporter with or without wildtype or dominant-negative (H53A) RNF146 expression constructs. (D) qRT (quantitative real-time)-PCR mRNA expression analysis of Wnt target genes in PA-1 cells transfected with RNF146, LRP6 (positive control), and non-targeting (negative control) siRNAs. Cells were either unstimulated with exogenous Wnt3a (blue) to test effects on autocrine Wnt signaling [18], or further induced with Wnt3a (red).

To identify new genes mediating Wnt signaling, we screened 653 siRNA pools targeting the complete set of predicted ubiquitin E3 ligases, as well as a small group of zinc finger proteins of unknown function. The screen was conducted in a HEK293 cell line stably expressing different luciferase reporters driven by either TCF binding sites or the SV40 promoter, and purified Wnt3a protein was used to stimulate signaling [17]. A Z-score cutoff of -1.65 was used to identify the top 5.7% of screen hits (37 targeted genes) that reduce Wnt reporter activity (normalized to SV40 reporter activity), and these hits were further limited to 26 genes that did not significantly affect SV40 luciferase activity (Z-score less than 1.7; Figure 1A). When individual siRNAs were tested, four genes, including RNF146, showed at least 3 of 4 siRNAs producing greater than two-fold reduction of Wnt signaling in HEK293 cells (Figure 1B and Table S1). Doxycycline-inducible RNF146 miRNA expression also inhibits Wnt signaling, and this effect is rescued by RNF146 overexpression (Figure 1C). We tested RNAi of these genes for effects on endogenous Wnt target gene expression in PA-1 teratocarcinoma cells, which display autocrine Wnt signaling [18], and found that multiple siRNAs for only RNF146 inhibit expression of Wnt-activated genes GAD1 and SAX1, as well as enhance expression of Wnt-repressed genes LEFTY1 and LEFTY2 (Figure 1D).

### RNF146 Destabilizes Axin and Cooperates with Tankyrase

**Figure 2:**
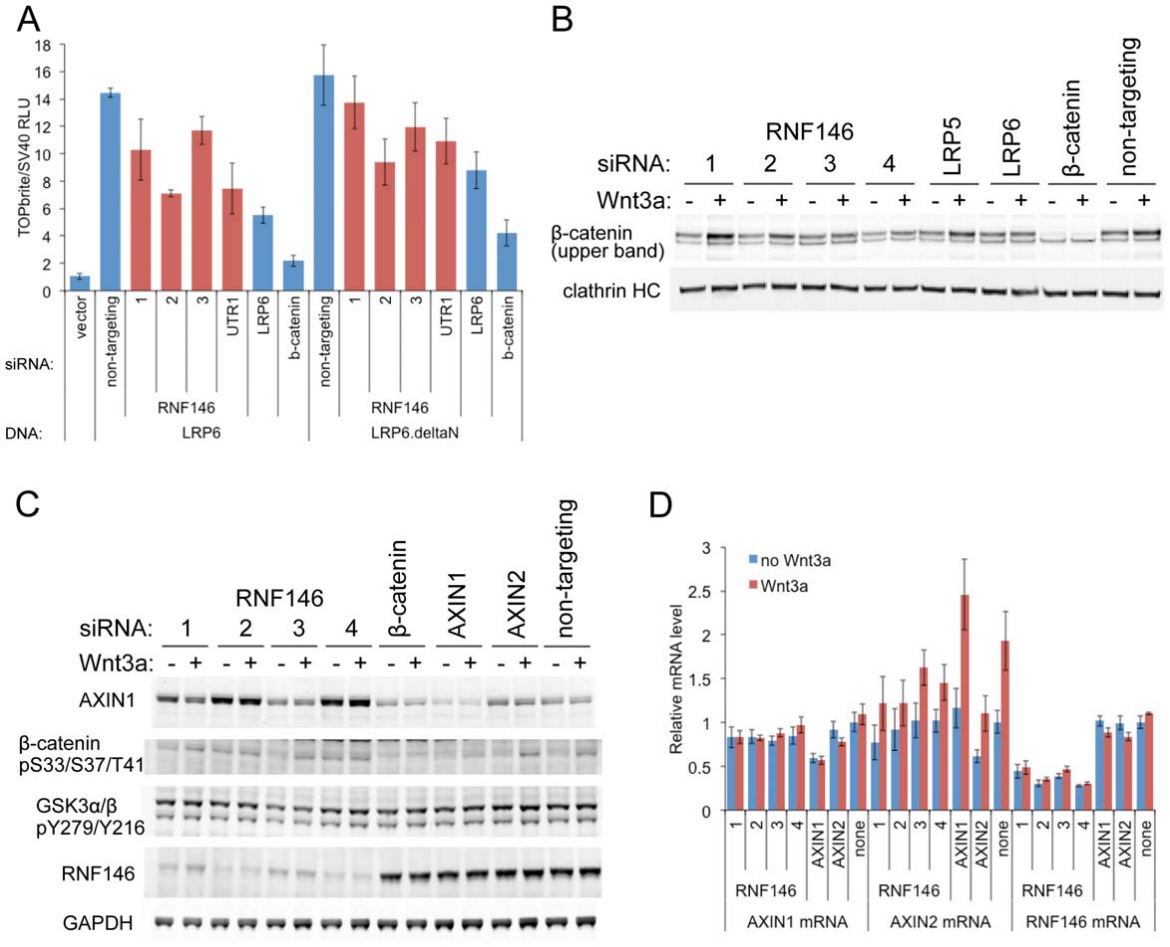
RNF146 acts in the Wnt pathway at the level of Axin protein destabilization. (A) Effects of individual RNF146 (red) and control (blue) LRP6, β-catenin, or non-targeting siRNAs on Wnt reporter activity in HEK293 cells stably expressing TOPbrite and SV40 reporters, and transiently transfected with expression construct for either wildtype LRP6 or a constitutively active LRP6 with the extracellular domain deleted (deltaN). (B) Western analysis of the effects of individual RNF146 siRNAs on β-catenin stabilization induced by Wnt3a (+) in HEK293 cells. LRP5 and LRP6 siRNAs are used as negative and positive controls, respectively, since Wnt signaling in HEK293 cells depends on LRP6 and not LRP5 [17]. β-catenin siRNA indicates that the higher molecular weight band in the panel corresponds to β-catenin protein. Clathrin heavy chain immunoblotting was used as a gel loading control. (C) Western analysis of AXIN1, RNF146, β-catenin phosphoshorylated on Ser33, Ser37, and Thr41, and active GSK3α/β auto-phosphorylated on Tyr279/216 after transfection of individual RNF146 siRNAs in HEK293 cells with (+) or without (−) Wnt3a stimulation. β-catenin and AXIN1 RNAi confirm the specificity of the antibodies. GAPDH was used as a loading control. (D) qRT-PCR analysis of AXIN1, AXIN2, and RNF146 mRNA after transfection of individual RNF146 siRNAs in HEK293 cells. Note that AXIN2 expression is weakly induced by Wnt3a (red), and this response is regulated by the RNAi treatments.

To define the level in the Wnt pathway at which RNF146 functions, we stimulated signaling by overexpressing Wnt coreceptor LRP6 or a constitutively active form of LPR6 in HEK293 cells [19], and found that RNF146 siRNAs reduce signaling (Figure 2A). RNF146 RNAi also inhibits Wnt3a-stimulated β-catenin stabilization (Figure 2B) and, at least in the absence of Wnt induction, promotes β-catenin phosphorylation at sites that mediate its degradation (Figure 2C). We next examined the effects of RNF146 RNAi on proteins that act in the Wnt pathway between LRP6 and β-catenin, and found that AXIN1 and AXIN2 steady-state levels are dramatically increased, whereas total and phospho-GSK3α and -GSK3β levels are not altered (Figure 2C). RNF146 functions to destabilize Axin with or without induction of signaling by Wnt3a (Figure 2C), and this appears to be a post-transcriptional effect, since RNAi does not increase AXIN1 or AXIN2 mRNA (Figure 2D).

**Figure 3:**
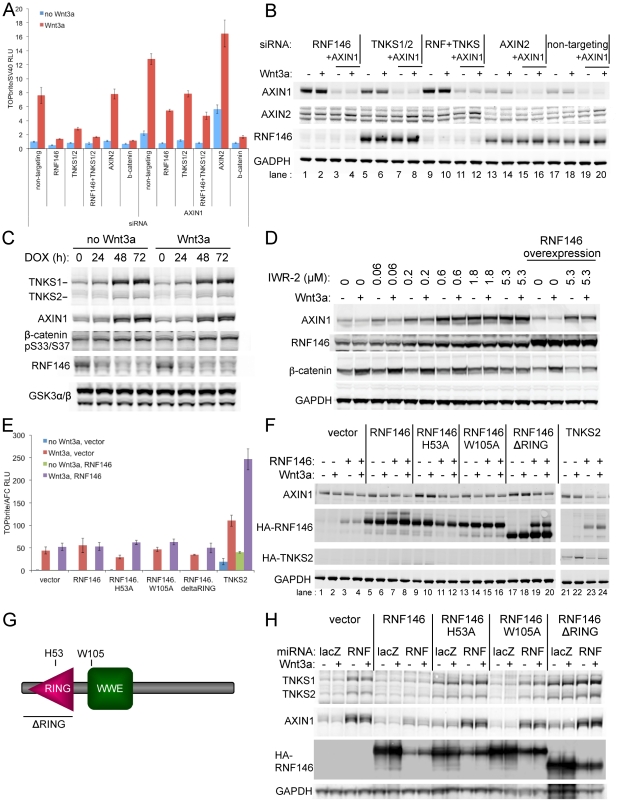
RNF146 and tankyrase function coordinately in Wnt signaling and regulate each other's protein stability. (A) Effects on Wnt signaling in the HEK293 reporter stable cell line with and without Wnt3a stimulation for siRNAs targeting RNF146, the combination of tankyrases TNKS1 and TNKS2, or the combination of all three genes. On the right half of the graph, these siRNAs are also combined with AXIN1 siRNA. AXIN2, β-catenin, and non-targeting siRNAs serve as controls. (B) Western analysis of whole cell lysates for HEK293 cells treated as in (A). (C) Time course of tankyrase (TNKS1 and TNKS2) and Axin stabilization in HEK293T cells stably expressing RNF146 miRNA that was induced for the indicated times with doxycycline (DOX), with or without Wnt3a added for the final 12 h of DOX induction. β-catenin phosphorylation and RNF146 protein knockdown levels are also shown. GSK3α/βimmunoblotting was used as a loading control. (D) Western analysis of HEK293 cells treated with tankyrase small-molecule inhibitor IWR-2 at the indicated concentrations for 16 h without (−) or with (+) Wnt3a induction. Whole cell lysates were monitored for AXIN1, RNF146, and β-catenin protein levels, and GAPDH serves as a loading control. Transgenic overexpression of RNF46 partially blocks AXIN1 protein stabilization by IWR-2 compound (right-most two lanes). (E) Wnt reporter activity in the HEK293 stable cell line transfected with expression constructs for the indicated RNF146 alleles, co-expressed with either control vector (blue, without Wnt3a stimulation, or red, with Wnt3a) or RNF146 (green, without Wnt3a, or purple, with Wnt3a). A cell viability assay was used to normalize Wnt luciferase reporter activity [17]. (F) Corresponding Western analysis of Axin, RNF146, and tankyrase proteins for cells treated as in (E). (G) Schematic representation of the RNF146 proteins produced by the expression constructs, with structural domains and mutation sites indicated. (H) Western analysis of endogenous tankyrase and Axin protein levels in HEK293T cells stably expressing doxycycline-induced RNF146 (RNF) or control (lacZ) miRNA after transfection with the indicated RNF146 or control vector expression plasmids, without (−) or with (+) Wnt3a induction. Anti-HA immunoblotting shows the expression level and RNAi-mediated knockdown of HA-RNF146 protein, and GAPDH levels serve as a loading control.

Since tankyrases TNKS1 and TNKS2 also positively regulate Wnt signaling by destabilizing Axin [9], we compared the effects of RNF146 and tankyrase RNAi in HEK293 cells. The level of Wnt signaling inhibition (Figure 3A) and AXIN1 or AXIN2 stabilization (Figure 3B) are similar for RNAi of RNF146 (lanes 1 and 2), tankyrases (both TNKS1 and TNKS2; lanes 5 and 6), or the combination of RNF146 and tankyrases (lanes 9 and 10), suggesting that RNF146 and tankyrase function together in a linear pathway rather than in parallel pathways that might have additive effects. This result is particularly evident in the presence of AXIN1 RNAi to enhance Wnt3a stimulation and limit the inhibitory effects of RNF146 and tankyrase RNAi to that resulting from only AXIN2 stabilization (Figure 3A), since AXIN1 stabilization is blocked by AXIN1 RNAi (Figure 3B, lanes 3, 4, 7, 8, 11, and 12). Surprisingly, we found that RNF146 RNAi also increases TNKS1 and TNKS2 protein levels (Figure 3C and S1). Just as for Axin destabilization, RNF146 destabilizes tankyrases independent of Wnt stimulation (Figure 3C) and at a post-transcriptional level (Figure S2). Proteasome inhibition can also stabilize tankyrase proteins, though less strongly than RNF146 RNAi (Figure S1). Temporally, effects on Axin, tankyrase, and β-catenin proteins coincide such that induction of RNF146 RNAi maximally stabilizes Axin and tankyrase proteins by 48 h, the same time at which enhanced β-catenin phosphorylation becomes apparent, and 24 h after RNF146 protein levels begin to decline (Figure 3C).

We generated an antibody against RNF146 protein, and show that tankyrase also destabilizes RNF146 protein. Combined TNKS1 and TNKS2 RNAi increases RNF146 steady-state protein levels (Figure 3B, lanes 5 and 6), but does not affect RNF146 mRNA levels (Figure S2). Tankyrase-mediated degradation of RNF146 protein is independent of Wnt stimulation in HEK293 cells (Figure 3B, lane 5) and requires the catalytic activity of tankyrases, since the IWR-2 small-molecule inhibitor of TNKS1 and TNKS2 [9], [20] also stabilizes RNF146 protein in a dose-dependent manner similar its stabilization of Axin protein (Figure 3D).

Overexpression of TNKS2 in HEK293 cells induces Wnt signaling in the absence of ligand, and also enhances signaling stimulated by Wnt3a protein (Figure 3E). In contrast, RNF146 overexpression does not affect signaling with or without Wnt3a stimulation, but it does further potentiate signaling in combination with TNKS2 overexpression. Combined overexpression of RNF146 and TNKS2 also reduces Axin protein levels (Figure 3F, lanes 23 and 24).

RNF146 is a predicted ubiquitin E3 ligase that contains a RING domain, presumably binding E2 ubiquitin conjugating enzymes, and a WWE domain [21], putatively binding to substrates (Figure 3G). Expression of mutant forms of RNF146 that either delete the RING domain (ΔRING) or replace the putative zinc-coordinating His53 with Ala (H53A) partially suppresses signaling in Wnt3a-stimulated HEK293 cells (Figure 3E) and stabilize Axin and tankyrase proteins with or without Wnt3a induction (Figure 3F, lanes 9, 10, 17, and 18, and 3H). The dominant-negative effects of these mutant RNF146 transgenes on Wnt signaling and Axin protein can be reversed by overexpression of wildtype RNF146 (Figure 3E and 3F, lanes 11, 12, 19, and 20), suggesting that the mutant RNF146 proteins may lack ligase activity and compete with wildtype protein for substrate binding. Expression of mutant RNF146 in which the evolutionarily conserved Trp105 in the WWE domain is replaced with Ala (W105A) did not show any of these dominant-negative effects (Figure 3E–H).

**Figure 4:**
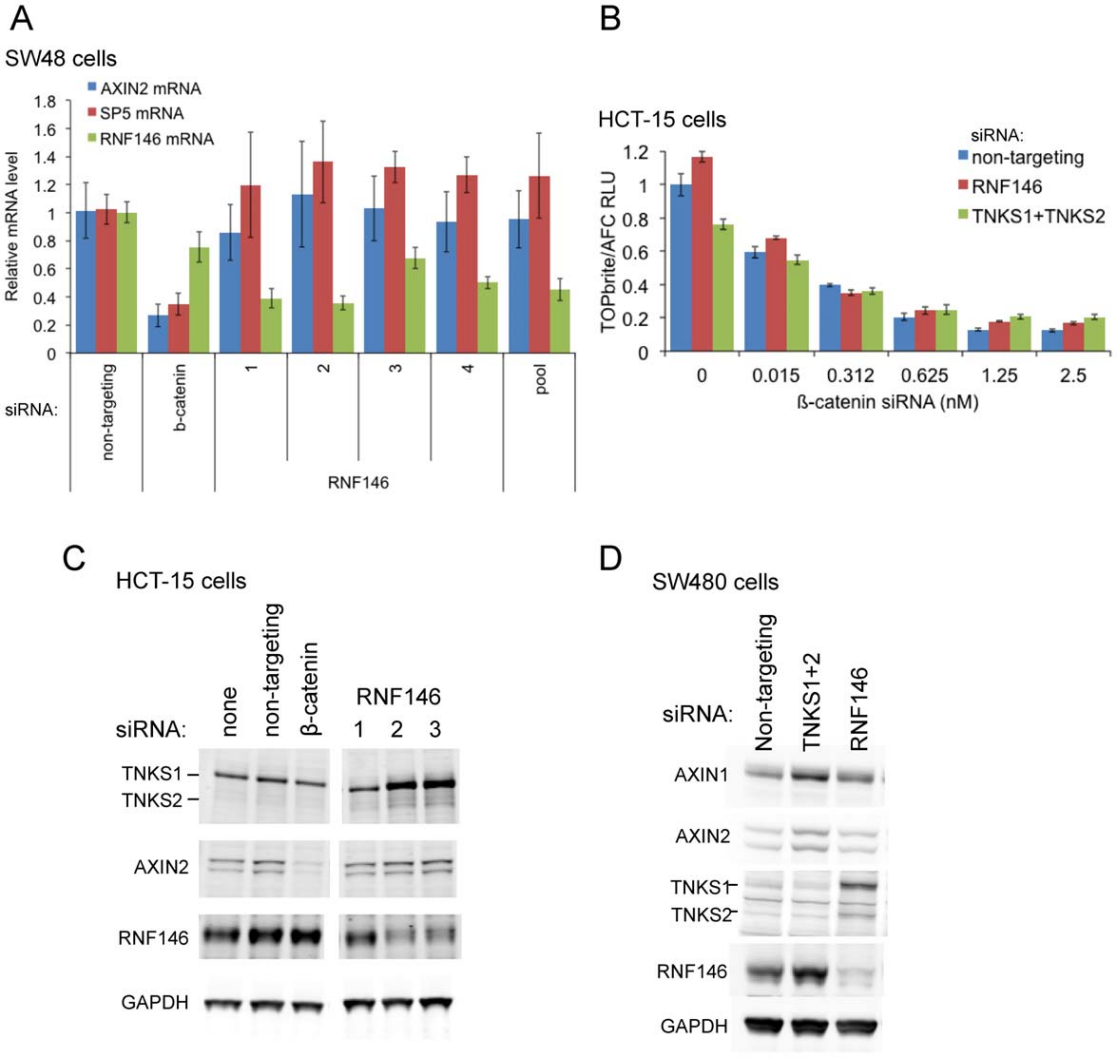
RNF146 RNAi does not inhibit Wnt signaling in β-catenin or APC mutant colorectal cell lines. (A) Expression levels of endogenous β-catenin-activated genes AXIN2 (blue) and SP5 (red) in SW48 cells after transfection of individual or pooled RNF146 siRNAs. β-catenin and non-targeting siRNAs serve as positive and negative controls, respectively, and the level of knockdown of RNF146 mRNA expression is indicated in green. (B) Co-transfection of RNF146 (red), tankyrase (both TNKS1 and TNKS2; green), or control non-targeting siRNA with β-catenin siRNA at the indicated concentration in HCT-15 cells stably expressing TOPbrite Wnt reporter. Reporter activity is normalized to cell number and non-targeting siRNA treatment alone. (C–D) Western analysis of tankyrase, Axin, and RNF146 protein levels in HCT-15 (C) or SW480 (D) cells after siRNA treatment targeting the indicated genes.

In colorectal cancer cell line SW48, Wnt signaling is activated by β-catenin mutation S33Y at a GSK3 phosphorylation site required for Axin-mediated β-catenin degradation [22]. As expected in these cells, RNF146 RNAi does not inhibit expression of β-catenin-activated genes AXIN2 and SP5 (Figure 4A). Colorectal cancer cell line HCT-15 is activated for Wnt signaling by an APC truncating mutation, and signaling is partially inhibited by tankyrase RNAi (Figure 4B). While RNF146 RNAi in HCT-15 cells can stabilize TNKS1 and TNKS2 proteins, it unexpectedly does not stabilize Axin (Figure 4C) or inhibit Wnt luciferase reporter activity (Figure 4B). RNF146 siRNAs have no effect on signaling even in combination with β-catenin siRNA at concentrations that only partially inhibit Wnt signaling. Lastly, in the colorectal cancer cell line SW480 that also possesses a truncating APC mutation, tankyrase RNAi stabilizes AXIN1, AXIN2, and RNF146 proteins (Figure 4D). In contrast, RNF146 RNAi in SW480 cells stabilizes tankyrase proteins but not AXIN1 or AXIN2.

### RNF146 Binds and Ubiquitylates Tankyrase and Axin

**Figure 5:**
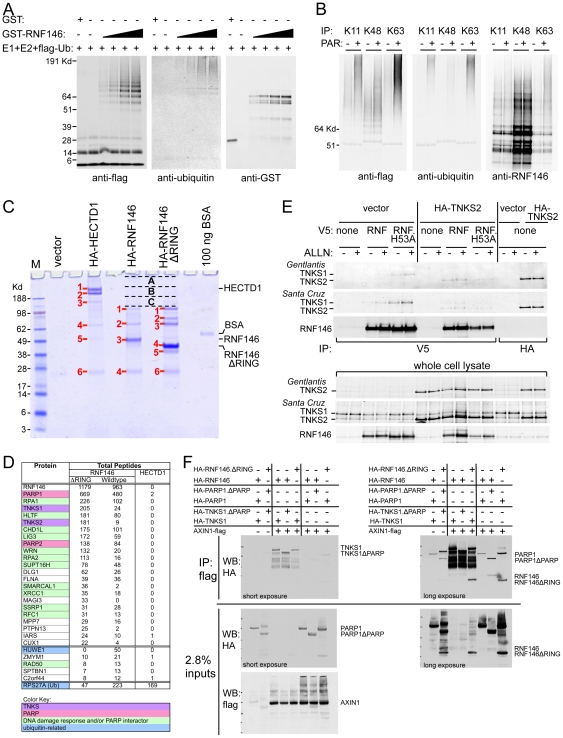
RNF146 displays ubiquitin E3 ligase activity *in vitro* and binds to tankyrase, PARP1, and Axin proteins. (A) Western analysis of auto-ubiquitylation reactions with variable amounts of GST-RNF146 protein (see Materials and Methods) immunoblotted for flag-ubiquitin, polyubiquitin, or GST. GST protein serves as a negative control. (B) Western analysis as in (A) for GST-RNF146 ubiquitylation reactions incubated in the absence (−) or presence (+) of poly(ADP-ribose) [PAR] and immunoprecipitated with antibodies specific for K11-, K48-, or K63-linked polyubiquitin. Immunoblotting with RNF146 antibody serves as a loading control. Note that the K48 linkage-specific antibody more efficiently immunoprecipitates ubiquitylated RNF146, although the polyubiquitin chains are shorter in length and therefore less readily detected by anti-flag or -ubiquitin immunoblotting. (C) Coomassie-stained gel of anti-HA immunoprecipitates from cells transfected with the indicated expression constructs for RNF146 or control E3 ligase HECTD1. Numbered protein bands and lettered high-molecular-weight bands were excised for mass spectrometric analysis. (D) Table of proteins identified from the mass spectrometric analysis showing total numbers of peptides identified for each protein, combined for all numbered protein bands in (C). Shown are 23 proteins with the highest number of total peptides identified by interaction with RNF146ΔRING protein, and with fewer than three peptides in the spectrometric analysis of HECTD1 protein interactors. The second set of 5 proteins in the table show the greatest numbers of peptides identified for the analysis of interactors with wildtype RNF146 protein, but not HECTD1 protein. The code for the coloring is explained in the legend. (E) Western analysis of anti-V5 immunoprecipitation from HEK293 cells co-transfected as indicated for expression of V5-tagged wildtype or H53A mutant RNF146 (RNF), HA-tagged TNKS2, or control vector in the presence (+) and absence (−) of proteasome inhibitor ALLN. Co-immunoprecipitation of endogenous TNKS1 and overexpressed TNKS2 was assessed with anti-TNKS1/2 antibodies from the indicated two sources. RNF146 immunoblotting, anti-HA immunoprecipitation, and whole cell lysates are shown as controls. (F) Western analysis of immunoprecipitation of flag-tagged AXIN1 expressed in HEK293 cells co-transfected with the indicated expression constructs for HA-tagged wildtype or deletion mutant alleles of RNF146, TNKS1, or PARP1. Short exposure to film of the anti-HA immunoblot detects co-immunoprecipitated TNKS1 proteins, whereas longer exposure reveals RNF146 and PARP1 proteins. Input whole cell lysates probed for HA and flag detection are shown as controls for expression of the indicated proteins.

We confirmed that RNF146 possesses E3 ligase activity by purifying recombinant GST-RNF146 fusion protein from *E. coli* and demonstrating that it can catalyze in a dose-dependent manner auto-ubiquitylation *in vitro* in combination with purified E2 (UBCH5C) and E1 (UBE1) enzymes (Figure 5A). Immunoprecipitation with antibodies specific for K11, K48-, or K63-linked polyubiquitin [23], [24] indicates that all three linkage types can be specified by RNF146 and UBCH5C *in vitro* (Figure 5B). Since we demonstrate below that RNF146 ubiquitylates tankyrase and Axin, and these proteins are known to be PARsylated, we added poly(ADP-ribose) [PAR] polymers into the reaction and found that PAR enhanced RNF146 auto-ubiquitylation with all three linkage types (Figure 5B).

We used the active GST-tagged RNF146 protein and GST control protein to affinity purify interacting proteins from cell lysates of three cell lines (HEK293, HEK293 activated for Wnt signaling by LRP6 overexpression, and HCT-15). Mass spectrometric identification of protein interactors revealed seven proteins that were identified by at least 10 unique peptides in all three cell lines by GST-RNF146, but were not identified in any of the cell lines using the GST control protein (Figure S3). These specific interacting partners include PARP1 and three other proteins involved in DNA damage response.

As a complimentary approach to identify RNF146 interacting proteins, we expressed in HEK293 cells RNF146 wildtype and ΔRING proteins, as well the control E3 ligase HECTD1, and immunoprecipitated these HA-tagged proteins from cell lysates. Mass spectrometric identification of proteins in all Coomassie-stained electrophoretic gel bands (Figure 5C) yielded TNKS1, TNKS2, PARP1, and PARP2 among the most prevalent proteins captured by RNF146ΔRING but not HECTD1, in addition to 11 other proteins known to bind PARP1/2 or function in DNA damage response (Figure 5D). Most of these protein interactors were also captured by wildtype RNF146 protein, although typically identified by fewer total peptides, suggesting that ubiquitylation by active RNF146 may release interactors from a complex. In contrast, specific interacting proteins that were identified by more peptides after capture by wildtype rather than ΔRING RNF146 include ubiquitin and the HECT-type ubiquitin E3 ligase HUWE1.

We confirmed by Western analysis that RNF146 binds tankyrase by immunoprecipitating tagged RNF146 expressed in HEK293 cells (Figure 5E). Both wildtype and H53A mutant RNF146 proteins co-immunoprecipitate with endogenous TNKS1, apparently the predominant tankyrase in HEK293 cells, and with overexpressed TNKS2. Note that proteasome inhibitor ALLN weakly stabilizes TNKS2 and RNF146 when both are overexpressed. While we did not identify Axin proteins in the RNF146 affinity purifications for mass spectrometric analyses, we were able to show that at least overexpressed AXIN1 associates with tagged RNF146 and tankyrase (Figure 5F). TNKS1 co-immunoprecipitates with AXIN1, and this interaction does not require the PARP catalytic domain of TNKS1. These complexes can also contain RNF146 or, more abundantly, RNF146ΔRING protein. Overexpressed PARP1, with or without the PARP catalytic domain, is not specifically co-immunoprecipitated with AXIN1, although a small amount is nonspecifically immunoprecipitated under these conditions, with longer photographic exposure time required for detection. RNF146 wildtype and ΔRING proteins can also be identified in the AXIN1 complexes at similar abundance with or without TNKS1 overexpression, suggesting that RNF146 may bind directly to Axin as well as tankyrase.

**Figure 6:**
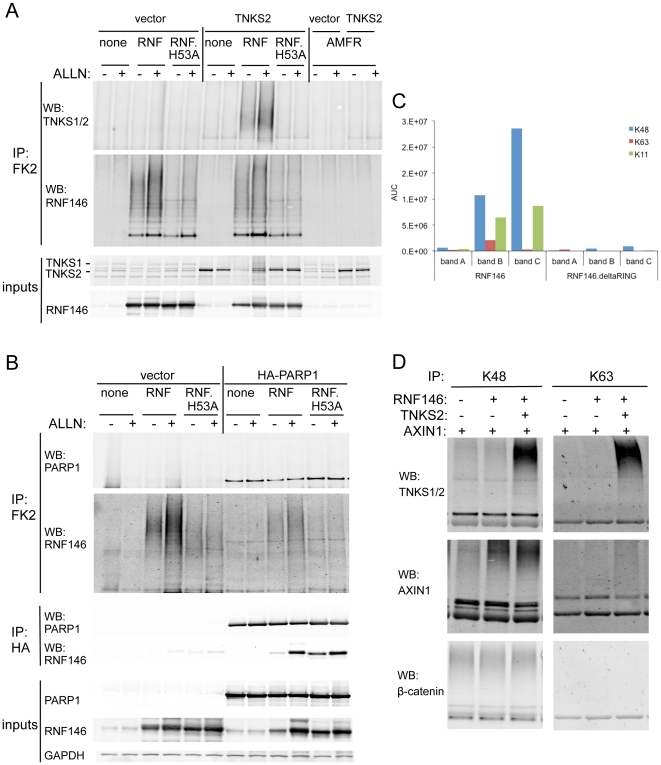
RNF146 ubiquitylates tankyrase and Axin in cells. (A) Western analysis of anti-ubiquitin (FK2 antibody) immunoprecipitation from HEK293 cells transfected with expression constructs for wildtype or H53A mutant RNF146 (RNF) or control E3 ligase AMFR, in combination with either TNKS2 or control vector DNA, without (−) or with (+) proteasome inhibitor ALLN treatment. Immunoblots are shown for ubiquitylated tankyrase and RNF146 smears. Input lysate immunoblots show expression levels of tankyrase and RNF146. (B) Western analysis analogous to (A) except that HA-tagged PARP1 is overexpressed rather that TNKS2. RNF146 binding to PARP1 is confirmed by anti-HA immunoprecipitation. (C) Relative quantitation of the area under the mass spectra curve (AUC) for K48-, K63-, and K11-linked polyubiquitin -GG signature peptides in excised gel bands A, B, and C depicted in Figure 5C. Results are shown for immunoprecipitation of wildtype or H53A mutant RNF146 proteins expressed in HEK293 cells. (D) Western analysis of immunoprecipitation with K48 or K63 linkage-specific polyubiquitin antibodies for the indicated overexpression of RNF146, tankyrase, or Axin in HEK293 cells. Immunoblotting for tankyrase, Axin, or control β-catenin proteins detects high-molecular-weight polyubiquitylated protein species.

Since RNF146 interacts robustly with tankyrase in biochemical and genetic assays, we tested whether RNF146 ubiquitylates tankyrase in cells. Affinity purification of HEK293 cell lysate with the FK2 anti-polyubiquitin antibody, which recognizes multiple ubiquitin linkage types, does not allow detection of endogenous tankyrase or RNF146 by Western analysis, but overexpressed RNF146 shows high-molecular-weight polyubiquitylated species (Figure 6A). Since mutant RNF146^H53A^ protein displays much less ubiquitylation, most of the ubiquitylation of wildtype RNF146 in cells is likely mediated by auto-ubiquitylation. TNKS2 overexpression alone or in combination with RNF146^H53A^ yields little or no polyubiquitylated tankyrase, however co-expression with wildtype RNF146 dramatically ubiquitylates TNKS2. Proteasome inhibitor ALLN partially attenuates RNF146-mediated degradation of total tankyrase protein levels while stabilizing TNKS2 that is polyubiquitylated by RNF146, suggesting that at least some of the ubiquitylated tankyrase is normally targeted to the proteasome. These effects seem to result from specific interactions between RNF146 and tankyrase. Neither tankyrase degradation nor ubiquitylation is observed with overexpression of a different RING-type E3 ligase, AMFR (Figure 6A). Although overexpressed RNF146 binds co-expressed PARP1, it does not mediate its ubiquitylation or destabilization (Figure 6B).

To define the ubiquitin linkage types specified by RNF146 on itself or on substrate proteins in cells, we analyzed the high-molecular-weight gel bands of the cell lysates immunoprecipitated for HA-RNF146 wildtype and ΔRING proteins (Figure 5C) for mass spectrometric peptide signatures of K48-, K63-, or K11-linked polyubiquitin [25]. All three ubiquitin linkage types were identified in the RNF146, but not RNF146ΔRING, immunoprecipitate (Figure 6C). Additional mass spectrometric analyses of these high-molecular-weight fractions revealed that the only proteins displaying appreciably more peptide spectral masses in the RNF146 bands than in the RNF146ΔRING bands were ubiquitin, RNF146, and HUWE1 (data not shown). This suggests that the primary carrier of ubiquitin in these bands is RNF146 and, to a lesser extent, HUWE1. Presumably other ubiquitylated substrates are not observed in the wildtype RNF146 immunoprecipitate because they dissociate from the ligase complex upon ubiquitylation.

To directly analyze the ubiquitin linkage types ligated onto substrate proteins by RNF146, we overexpressed RNF146 with tankyrase or Axin in HEK293 cells and immunoprecipitated polyubiquitylated proteins with antibodies specific for K48- or K63-linked polyubiquitin (Figure 6D). RNF146 strongly induced both K48- and K63-linked ubiquitylation of TNKS2 protein, whereas AXIN1 showed predominantly K48-linked ubiquitin. As a control for specificity, β-catenin in these immunoprecipitates displayed only K48-linked ubiquitin and was not altered by RNF146 overexpression.

### RNF146 Regulates Tankyrase Subcellular Localization

**Figure 7:**
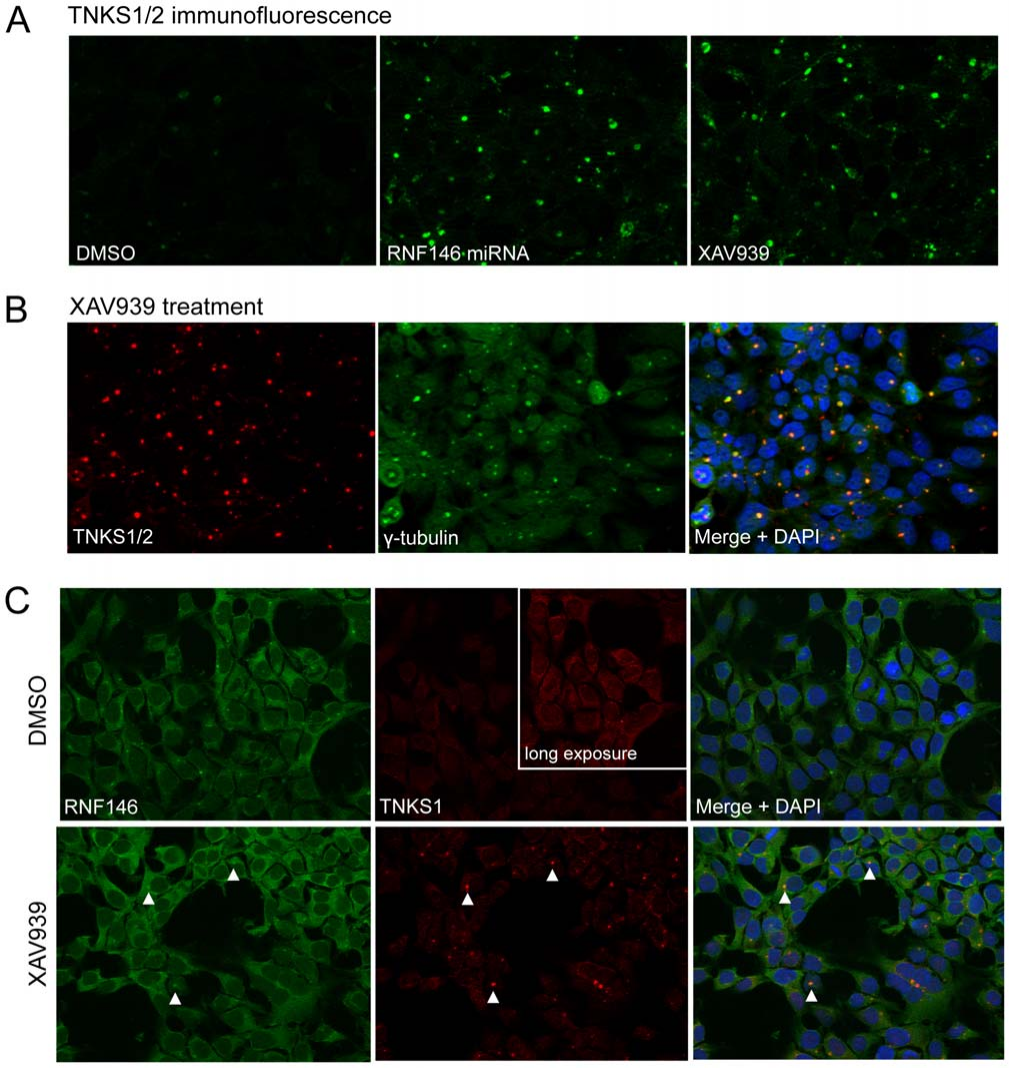
RNF146 RNAi and tankyrase inhibition induce tankyrase puncta in cells. (A) Immunofluorescence imaging of tankyrase (green) in HEK293T cells stably expressing doxycycline-inducible RNF146 miRNA treated with either control DMSO, doxycycline (RNF146 miRNA), or tankyrase inhibitor XAV939. (B) HEK293 cells treated with XAV939 and immunostained for endogenous tankyrase (red) and γ-tubulin (green). The merged image shows co-localized tankyrase with γ-tubulin (yellow) counterstained for nuclei with DAPI (blue). All images are representative of at least three independent experiments. (C) HEK293 cells were treated with DMSO or XAV939 and immunostained for endogenous RNF146 (green) and tankyrase (red), with DAPI counterstaining (blue) in the merged image. Arrowheads indicate co-localization of RNF146 and tankyrase in puncta.

We used immunocytochemistry to determine the fate of tankyrase protein that is stabilized upon RNF146 miRNA expression in HEK293 cells. Antibody that detects both TNKS1 and TNKS2 shows staining that is generally faint, diffuse, and uniform throughout interphase cells, with occasional puncta (Figure 7A) [26]. In sharp contrast, RNF146 protein depletion by RNAi produces intensely staining puncta of tankyrase protein. Treating cells with tankyrase small-molecule inhibitor XAV939 [9] similarly produces tankyrase puncta, typically a single large punctum or several smaller puncta in each cell. A portion of TNKS1 is known to localize to Golgi [27], however the localization of the Golgi marker protein giantin does not resemble the tankyrase puncta and is not altered by RNF146 RNAi (data not shown). During mitosis, but not interphase, tankyrase localizes to centrosomes [28], and we find that the single, large tankyrase punctum induced by XAV939 treatment in individual interphase cells colocalizes with γ-tubulin, indicating centrosomal localization (Figure 7B). In contrast to tankyrase, PARP1 shows exclusively nuclear localization that is not altered by XAV939 treatment (Figure S4).

The RNF146 rabbit monoclonal antibody shows diffuse cytoplasmic staining of HEK293 cells, with a single punctum also present in many cells (Figure 7C and S4), and this staining pattern is eliminated by RNF146 RNAi (data not shown). XAV939 treatment increases the level of diffuse cytoplasmic RNF146 protein, but does not seem to alter the punctate staining. A mouse monoclonal antibody against TNKS1, which detects tankyrase diffuse and punctate expression less readily but is compatible with the RNF146 antibody for double labeling, suggests that the RNF146 puncta colocalize with tankyrase puncta in XAV939-treated cells (Figure 7C).

## Discussion

**Figure 8:**
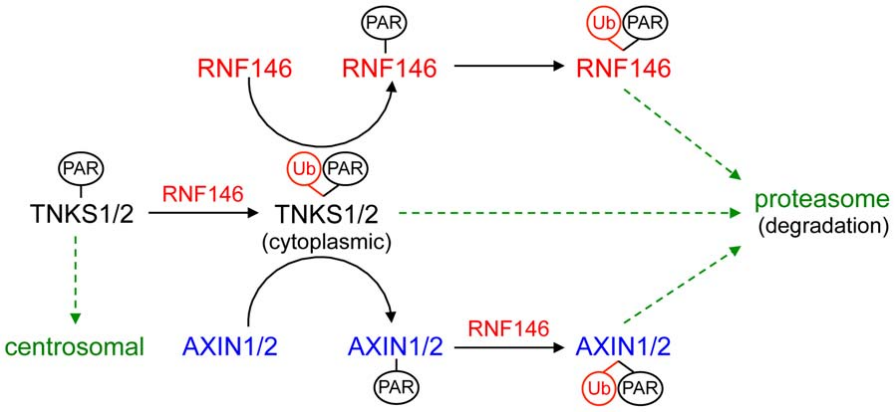
Model of RNF146 activity that leads to degradation of tankyrase, Axin, and RNF146 proteins. See Discussion for details. Green arrows indicate protein relocalization. “Ub” indicates ubiquitylation and “PAR” indicates PARsylation of the indicated proteins.

By screening a siRNA library targeting all human ubiquitin E3 ligases, we have identified RNF146 as a new positive regulator of Wnt signaling. RNF146 acts with tankyrase in the constitutive turnover of Axin proteins that maintains low levels of Axin and allows the Wnt receptor-coreceptor complex to initiate signaling by further repressing Axin's function in degrading β-catenin. Paradoxically, RNF146 also destabilizes through proteasomal degradation the tankyrase proteins, which are also positive regulators of Wnt signaling [9]. Upon RNF146 RNAi, the stabilized tankyrase protein relocalizes to a centrosomal location, which might represent aggregated and inactive protein in aggresomes [29]. Transgenic overexpression of tankyrase does not result in centrosomal puncta ([26], [30], and data not shown) however, interestingly, a tankyrase small-molecule inhibitor induced a similar stabilization and relocalization of tankyrase protein, indicating that PARsylation and ubiquitylation act together to maintain both proteasomal degradation and cytoplasmic localization of tankyrase (Figure 8). Since RNF146 seems to be localized to centrosomes, as well as the cytoplasm, this may be a site of tankyrase ubiquitylation and degradation, as it can be for misfolded proteins [29].

RNF146 protein is reciprocally destabilized by tankyrase activity, indicating that PARsylation and ubiquitylation target all three proteins in the RNF146-tankyrase-Axin complex to the proteasome for degradation. Since tankyrase inhibition prevents Axin ubiquitylation [9], PARsylation seems to be a signal for subsequent ubiquitylation, as has been proposed for proteasomal degradation of tankyrase substrate TRF1 [15] and tankyrase itself [16]. Indeed, purified PAR stimulates RNF146 auto-ubiquitylation *in vitro*.

During the preparation of this manuscript, Zhang *et al*. reported an independent identification of RNF146 as a regulator or Wnt signaling [31]. Consistent with our findings, Zhang *et al*. propose that RNF146 ubiquitylates Axin that has been PARsylated by tankyrase to target Axin for proteasomal degradation. We significantly expand the mechanistic understanding of this branch of the pathway to include: (1) RNF146 regulation of tankyrase ubiquitylation, protein stability, and subcellular localization; (2) tankyrase regulation of RNF146 degradation; (3) polyubiquitin linkage types specified by RNF146 on tankyrase, Axin, and itself; (4) RNF146 regulation of autocrine Wnt signaling in teratocarcinoma cells, but not signaling activated by APC or β-catenin mutation in multiple colorectal cancer cell lines; and (5) specificity of RNF146 for ubiquitylating and regulating tankyrases and not the related PARP1 protein.

We show that RNF146 can operate as a single-subunit RING-type E3 catalyzing both ubiquitin-substrate ligation and ubiquitin chain elongation in combination with E1 and E2 enzymes *in vitro*, and that the RING domain is required for function *in vivo*. RNF146 directs the ubiquitylation of tankyrase, Axin, and itself, and the polyubiquitin chains specified are of multiple or mixed linkage types, containing at least K48 and K63 linkages. These different linkage types are consistent with RNF146 controlling both the proteasomal degradation and subcellular localization of tankyrase. We also identified the HECT-type ubiquitin E3 ligase HUWE1 (also known as ARF-BP1 or MULE) as a RNF146 interacting protein that depends on the RING domain of RNF146 for binding and seems to associate with polyubiquitylated RNF146. This suggests that HUWE1 may function as an E4 enzyme in ubiquitin chain elongation for RNF146 substrates. Interestingly, HUWE1 contains a putative ubiquitin-binding UBA domain and a WWE domain.

While RNF146 RNAi can inhibit autocrine Wnt signaling in teratocarcinoma cells and also stabilize tankyrase proteins in colorectal cancer cells with APC mutation-driven signaling, we find that RNF146 knockdown does not significantly affect Wnt signaling or Axin protein stabilization in the colorectal cell lines tested. In HCT-15 cells, tankyrase RNAi or small-molecule inhibitors increase Axin levels and partially block signaling, suggesting that there may be a functionally redundant ligase in some cell types. While there are other predicted E3s in the human genome with WWE domains, we find no clear RNF146 paralog. Testing combination RNAi of RNF146 and HUWE1 in colorectal cancer cells may be informative.

RNF146 can also bind PARP1 and PARP2, although apparently with lower affinity than for tankyrase association. PARP1 protein levels are not affected by RNF146 and, since PARP1 protein is localized to the nucleus [32] whereas RNF146 protein is cytoplasmic, RNF146 may not mediate degradation of all PARP family members or of all PARsylated proteins. It will be important to further define the specificity of RNF146 in regulating the activity of different PARP enzymes, as well as to determine whether the other functions of tankyrases in different subcellular locations, such as telomere elongation, are regulated by RNF146.

The function of RNF146 in mouse development or human disease is currently unknown. The chromosomal region of RNF146 has been linked to breast cancer risk in a Jewish Ashkenazi population, although no mutations in the protein coding region have been identified [33], [34]. While RNF146 is expressed in all human tissues examined in one study, expression was upregulated in the brain of Alzheimer's disease patients [35]. It will be interesting to specifically investigate a role for RNF146 in Wnt-dependent developmental and disease processes once mouse knockout strains have been generated.

## Materials and Methods

### Cell Culture and Reagents

HEK293, PA-1, SW480, SW48, and HCT-15 cell lines were purchased from the American Type Culture Collection. An HCT-15 cell line stably integrated with TOPbrite reporter [17] was selected for hygromycin resistance, and luciferase activity for these cells was normalized to AFC fluorescence with the CellTiter-Fluor cell viability assay (Promega).

cDNAs for human RNF146 (NM_030963), TNKS1 (NM_003747), TNKS2 (NM_025235), and PARP1 (NM_001618) were purchased from OriGene and subcloned into pRK mammalian expression vectors. The RNF146ΔRING allele deletes the region from Met1 through Cys82, TNKS1ΔPARP deletes the region from Ala1112 through Thr1327, and PARP1ΔPARP deletes Asp797 through Trp1023. Transfections were performed with FuGENE HD (Roche) according to the manufacturer's instructions. Plasmid transfections were assayed for expression at 48 h, except for detection of ubiquitylated proteins which were assayed after 20 h.

RNF146 protein was detected with immune sera from a rabbit immunized with GST-RNF146 protein produced in *E. coli*, or using a monoclonal antibody derived from this rabbit (Epitomics, Inc.). TNKS1 and TNKS2 were detected with rabbit antibody H-350 (Santa Cruz Biotechnology, Inc.) or mouse monoclonal antibody 19A449 (Genlantis). AXIN1 and AXIN2 were detected with rabbit monoclonal antibodies C95H11 and 76G6, respectively (Cell Signaling Technology). β-Catenin was detected with a mouse monoclonal antibody (BD Biosciences). Phosphorylated β-Catenin was detected with either rabbit anti-phospho-Ser33/Ser37/Thr41 (Cell Signaling Technology) or mouse monoclonal BC-22 anti-phospho-Ser33/Ser37 (Santa Cruz Biotechnology).

Wnt3a protein (R&D Systems) in a concentration range of 0.05 to 0.2 µg/ml was used for cell stimulation. Compounds XAV939 and IWR-2 were purchased from Tocris or synthesized *de novo* [20], respectively.

### RNAi Screening and Assays

Human E3 ligase siRNA pools (Dharmacon, Inc.) were reverse transfected in 96-well format at a final concentration of 25 ηM into HEK293 cells stably expressing Wnt and SV40 luciferase reporters [17]. After 72 h, cells were treated with Wnt3a-conditioned medium for 6 h, and firefly and Renilla luciferase activities were assayed as previously described [17]. Z-scores were calculated for normalized luciferase activities of each siRNA pool by determining the number of standard deviations from the mean of all siRNA pools in the screen. siRNA pools that deviated from the normal distribution for the SV40 control reporter were eliminated from consideration. The positive regulators targeted by siRNA pools for which the z-score was less than -1.65 were further evaluated by RNAi with individual siRNAs of the pool. Detailed data are available in Table S1.

ON-TARGETplus siRNAs and control siRNAs (Dharmacon) were used according to the manufacturer's instructions for transient RNAi. Individual or pools of 4 siRNAs were used at 10 ηM and 40 ηM, respectively. For PA-1, SW480, SW48, and HCT-15 cells, serial siRNA transfection was performed with cell passaging in between transfections to prolong target knockdown. BLOCK-iT inducible Pol II miR miRNA expression constructs and T-REx-293 cells (Invitrogen) were used according to the manufacturer's instructions to generate stable cell lines for RNAi induced by 2 µg/ml doxycycline. Targeting sequences used for RNAi are available in Table S2.

### qRT-PCR Expression Analysis

mRNA was isolated from cell lysates in an mRNA capture plate (Invitrogen) and assayed for specific transcripts using either FAM and TAMRA probes or FAM and non-fluorescent quencher probes depending on the target. Primers and probes were either purchased (Applied Biosystems) or custom designed (sequences available in Table S2). Primer-limited VIC/MGB GAPD (human Glyceraldehyde 3-phosphate dehydragenase) or VIC/MGB PGK (human Protein kinase, cGMP-dependent, type I) assays (Applied Biosystems) were used for normalization of mRNA levels to these endogenous controls. Reverse transcription and quantitative PCR was performed with one-step RT-PCR mix (Applied Biosystems) and run on a 7900 HT real time PCR instrument (Applied Biosystems). Relative mRNA values were calculated by the ΔΔCt method, normalizing to GAPDH or PGK mRNA.

### Western Immunoblot Analysis

Cell lysates were prepared in lysis buffer: 20 mM Tris pH 7.5, 135 mM NaCl, 1% IGEPAL ca-630, 0.5% n-Dodecyl-ß-maltoside, 10% glycerol, 1.5 mM MgCl_2_ and Complete EDTA-free Protease Inhibitor and Phosphatase Inhibitor (Roche). To isolate soluble β-catenin, cells were lysed in Passive Lysis Buffer (Promega) supplemented with Complete EDTA-free Protease Inhibitor Cocktail (Roche). To examine ubiquitylated proteins, denatured lysates were obtained using lysis buffer containing 7 M urea and 2 mM NEM. Proteasome inhibition was performed by treating cells for 4 h with 10 µM N-Acetyl-Leu-Leu-Nle-CHO (ALLN; EMD Chemicals). Protein concentration was measured using a standard BCA assay (ThermoFisher). 15 µg of protein in cell lysate was loaded for polyacrylamide gel electrophoresis. Highly cross-adsorbed goat anti-rabbit or anti-mouse IgG conjugated to Alexa Fluor 680 (Invitrogen) or infrared DYE 800 (Rockland) was used in combination for dual labeling and secondary detection. Western blots were scanned using the LI-COR Odyssey imager. Raw images were processed to visualize the linear range with Adobe Photoshop software.

Immunoprecipitations were performed with 50 µg of protein in cell lysates for detection of overexpressed proteins, and with up to 500 µg for endogenous protein detection. Immunoprecipitation for mass spectrometry or ubiquitylated proteins was performed with 400 µg to 10 mg of protein in cell lysate. Anti-V5 agarose (Sigma), anti-gD monoclonal antibody 952 (Genentech, Inc.), anti-HA3F10 affinity matrix (Roche), and glutathione sepharose 4B (GE Healthcare) were used for affinity purification of V5-, gD-, hemagglutinin (HA)-, and glutathione S-transferase (GST)-tagged proteins, respectively.

### Ubiquitylation Assays

*In vitro* ubiquitylation was performed in 100 mM Tris pH 7.4, 100 mM MgCl_2_, 100 mM DTT, 100 mM NaF, 20 µM okadaic acid, 1 mM ATP. A 30 µl reaction included 0.5 µg flag-ubiquitin, 0.5 µg untagged ubiquitin, 300 ηg UBE1 (BIOMOL International), 500 ηg UBCH5C (BostonBiochem), and variable amounts of GST-RNF146 (1.5, 3, 4.5, or 6 µg). Reactions were incubated at 30°C for 30 minutes or run to completion for 2 h. Poly(ADP-ribose) polymer (Trevigen) was added at a 1∶100 dilution of the commercial stock.

For immunoprecipitation of ubiquitylated proteins, 50 µl of a 50% slurry of polyubiquitin monoclonal FK2-agarose (MBL) was mixed with 400 µg of cell lysate. For immunoprecipitation with polyubiquitin linkage-specific antibodies, 10 µg of monoclonal 2A3/2E6 anti-K11, Apu2.07 anti-K48, or Apu3.A8 anti-K63 [23], [24] were each incubated with 3 mg of cell lysate, precipitated with Protein A plus beads (ThermoFisher), and washed as previously described [23], [24].

### Mass Spectrometry

Immunoprecipitation was performed as described above from 10 mg of cell lysate using 0.5 mg of GST-RNF146 or GST protein produced in *E. coli*. HA-tagged wildtype and ΔRING RNF146 proteins expressed in HEK293 cells were isolated from 3.5 mg of cell lysate with anti-HA 3F10 affinity matrix (Roche). Affinity purified proteins were concentrated, reduced in sample buffer (Sigma) at 90°C for 5 min, and alkylated in 0.176 M n-iospropyl iodoacetamide at room temperature for 20 min. Samples were then separated by 4–20% Tris-Glycine SDS-PAGE (Invitrogen). The gel was stained with Coomassie Blue, and gel bands were excised (spanning the entire gel lanes for GST affinity purifications), destained, and dehydrated as previously described [25]. Proteins were digested with trypsin (Promega) in ammonium bicarbonate pH 8 overnight at 37°C. Peptides were extracted from the gel slices, dried, and subsequently reconstituted in 2% acetonitrile and 0.1% formic acid.

Samples were injected onto a 100 µM×100 mm column (BEH, 1.7 µM, Waters Corp) at a flow rate of 1 µL/min using a NanoAcquity UPLC (Waters Corp) and peptides introduced into a hybrid LTQ-Orbitrap mass spectrometer (ThermoFisher) via nanospray ionization, as previously described [25]. Data was collected in data-dependent mode with the parent ion being analyzed in the FTMS and the top 8 most abundant ions being selected for fragmentation and analysis in the LTQ. Tandem mass spectrometric data was analyzed using the Mascot search algorithm (Matrix Sciences). For examining polyubiquitin linkages, the peak areas for ubiquitin -GG signature peptides were extracted within a 10 ppm window using a label free approach [25].

### Immunofluorescence Imaging

HEK293T cells integrated with RNF146 miRNA expression construct were seeded in chamber slides and induced for RNAi with 2 µg/ml doxycycline for 48 h, or treated with DMSO or 5 µM XAV939 for 16 h. Cells were fixed directly in culture medium with 4% paraformaldehyde in 0.075% saponin, then washed and permeabilized with 0.5% Saponin in Dulbecco's PBS. Rabbit anti-TNKS H-350 (Santa Cruz Biotechnology) and mouse anti-γ-tubulin GTU-88 (Abcam) antibodies were used for subcellular localization studies. Mouse monoclonal anti-tankyrase 6D790 and BL-2 (Santa Cruz Biotechnology) antibodies were used for co-localization studies with rabbit monoclonal anti-RNF146 antibody (described above). Secondary detection was with highly cross-adsorbed goat anti-mouse or anti-rabbit IgG conjugated to either ALEXA 488 or ALEXA 555. Slides with coverslips were mounted in Prolong Gold containing 4′,6-diamidino-2-phenylindole (DAPI; Invitrogen). Images were captured with a Zeiss MRm camera mounted on a Zeiss Axiovert 200M equipped with the ApoTome system. Optical sections were acquired with Axiovision software V4.8.2, and slices were summed using ImageJ (NIH) and combined using Adobe Photoshop Software. For weak tankyrase staining (inset image in Figure 7C) the brightness was adjusted using the auto levels setting without changing the gamma level.

## Supporting Information

Figure S1 **Tankyrase, Axin, and RNF146 proteins are weakly stabilized by proteasome inhibition.** Western analysis of tankyrase, RNF146, and Axin protein levels in HEK293 cell lines stably expressing doxycycline (Dox)-inducible miRNA targeting either RNF146 or lacZ (control). miRNA expression was induced by Dox treatment (+) and proteasome activity was inhibited with 20 µM MG132 for 2 h.
(TIF)

Figure S2 **RNF146 and tankyrase RNAi do not significantly inhibit Wnt signaling in SW480 colorectal cells.** qRT-PCR mRNA expression analysis of Wnt target genes AXIN2 (blue), TNKS1 (red), TNKS2 (green), and RNF146 (purple) in SW480 cells transiently transfected with siRNAs targeting either RNF146 or the combination of TNKS1 and TNKS2. A non-targeting siRNA serves as a control for normalizing mRNA levels.
(TIF)

Figure S3 **Affinity purification of putative RNF146 substrates with GST-tagged protein.** Proteins identified by mass spectrometry from affinity purification of cell lysates with either GST-RNF146 or control GST protein are listed, ranked by number of unique peptides identified from HEK293 cells. All proteins are shown that meet the following criteria: (1) identification in lysates from all three cell lines tested using GST-RNF146; (2) no identification in any of the three cell lines using GST protein; (3) identification by at least 10 unique peptides in HEK293 cells. Color coding is as described for Figure 5D.
(TIF)

Figure S4 **RNF146 RNAi does not affect PARP1 subcellular localization.** HEK293 cells were treated with DMSO or XAV939 and immunostained for endogenous RNF146 (green) and PARP1 (red). DAPI counterstaining shows nuclear PARP1 in the merged image (magenta).
(TIF)

Table S1 **E3 ligase Wnt pathway primary and secondary screen data.**
(XLS)

Table S2 **Sequences used for RNAi and qRT-PCR.**
(XLS)
