# Supplementary figures and images for: Ubiquitin Ligase RNF146 Regulates Tankyrase and Axin to Promote Wnt Signaling
Source: PLoS One. 2011 Jul 25;6(7):e22595. doi: 10.1371/journal.pone.0022595 (PMC3143158; doi:10.1371/journal.pone.0022595)

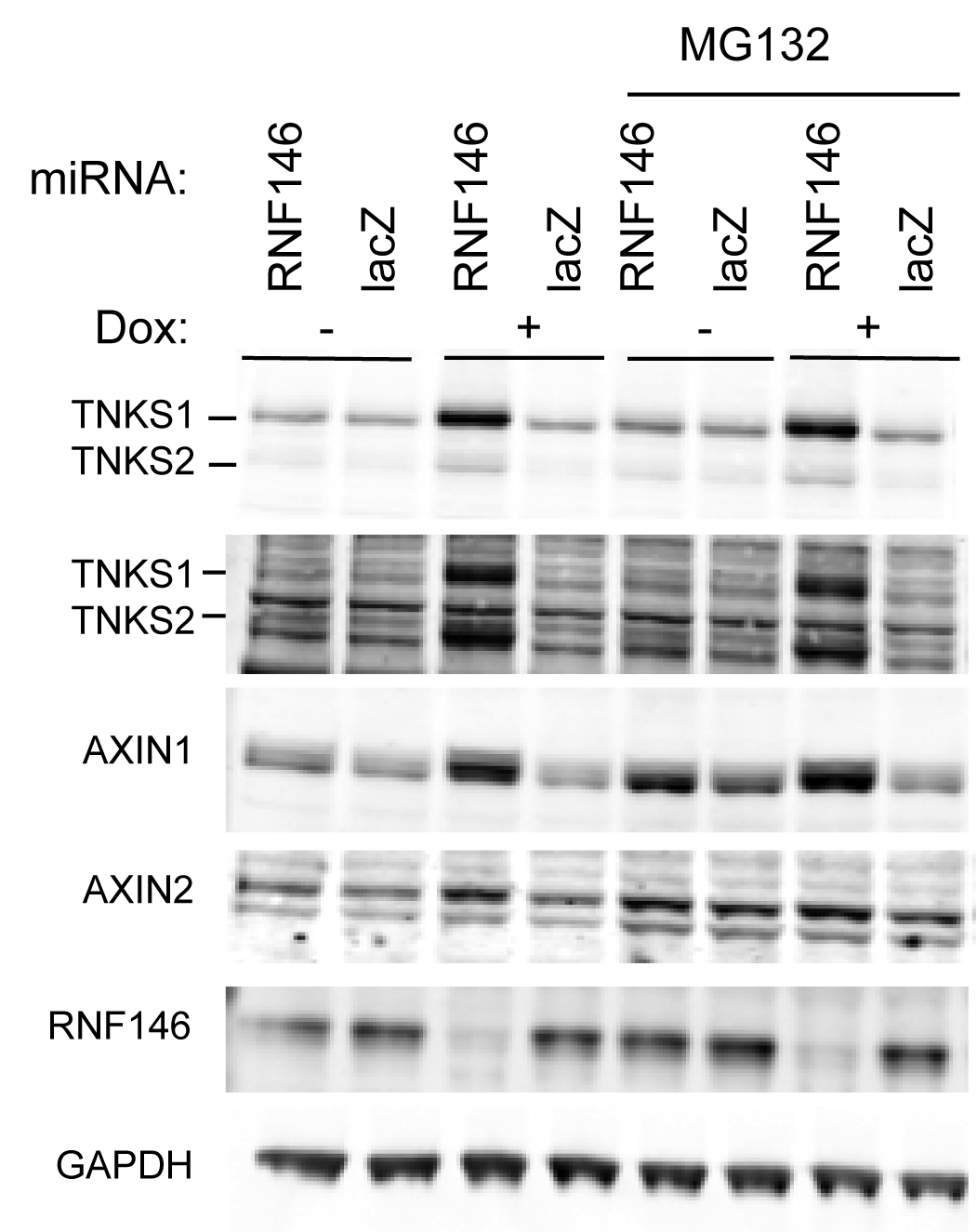

Supplement: Figure S1 — Tankyrase, Axin, and RNF146 proteins are weakly stabilized by proteasome inhibition. Western analysis of tankyrase, RNF146, and Axin protein levels in HEK293 cell lines stably expressing doxycycline (Dox)-inducible miRNA targeting either RNF146 or lacZ (control). miRNA expression was induced by Dox treatment (+) and proteasome activity was inhibited with 20 µM MG132 for 2 h. (TIF) [file pone.0022595.s001.tif]

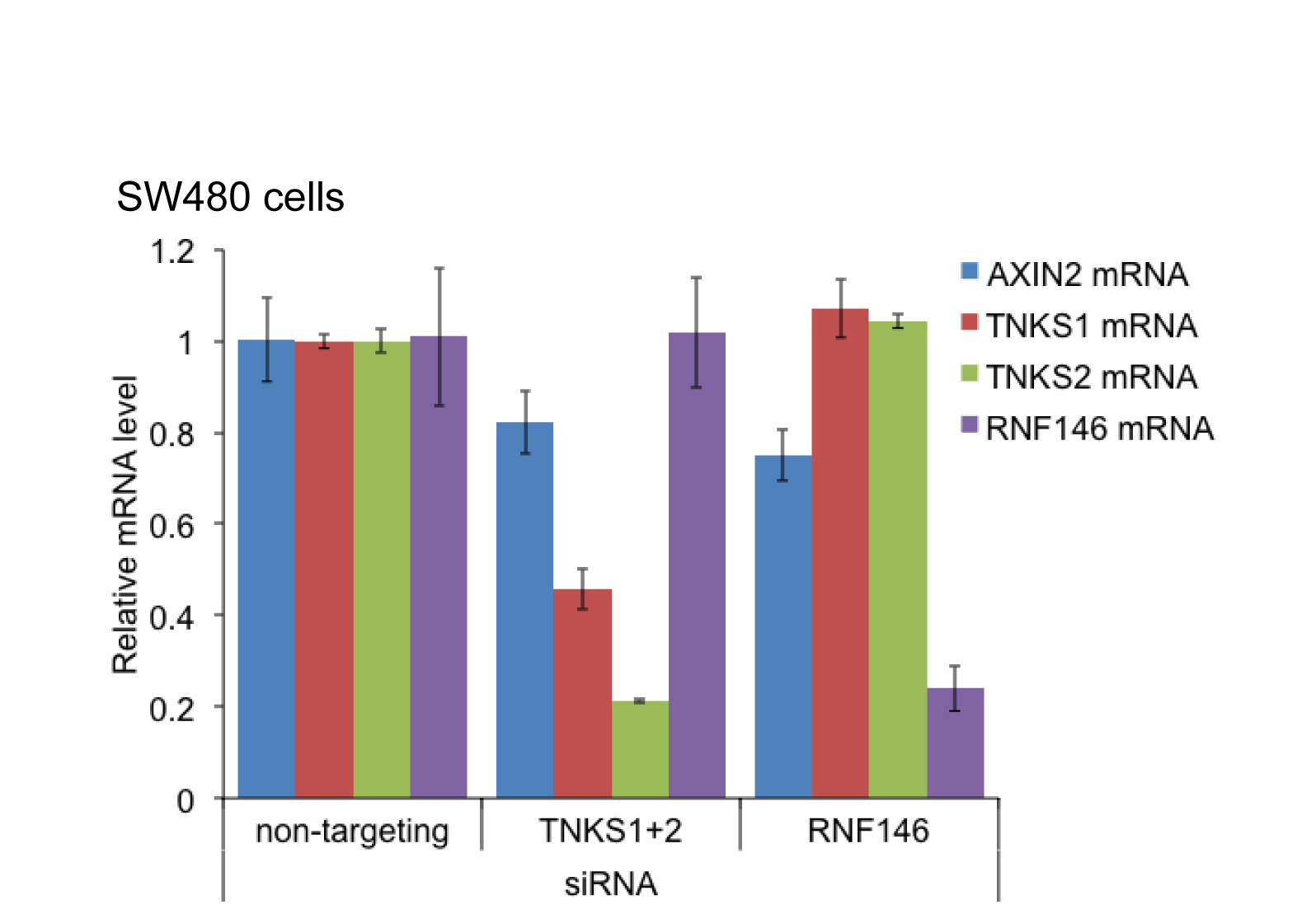

Supplement: Figure S2 — RNF146 and tankyrase RNAi do not significantly inhibit Wnt signaling in SW480 colorectal cells. qRT-PCR mRNA expression analysis of Wnt target genes AXIN2 (blue), TNKS1 (red), TNKS2 (green), and RNF146 (purple) in SW480 cells transiently transfected with siRNAs targeting either RNF146 or the combination of TNKS1 and TNKS2. A non-targeting siRNA serves as a control for normalizing mRNA levels. (TIF) [file pone.0022595.s002.tif]

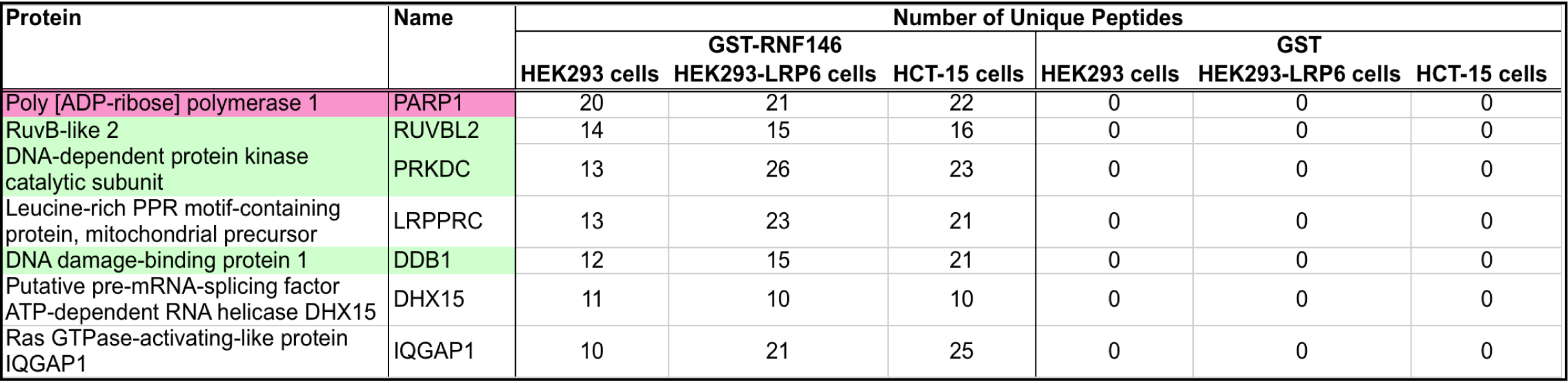

Supplement: Figure S3 — Affinity purification of putative RNF146 substrates with GST-tagged protein. Proteins identified by mass spectrometry from affinity purification of cell lysates with either GST-RNF146 or control GST protein are listed, ranked by number of unique peptides identified from HEK293 cells. All proteins are shown that meet the following criteria: (1) identification in lysates from all three cell lines tested using GST-RNF146; (2) no identification in any of the three cell lines using GST protein; (3) identification by at least 10 unique peptides in HEK293 cells. Color coding is as described for Figure 5D. (TIF) [file pone.0022595.s003.tif]

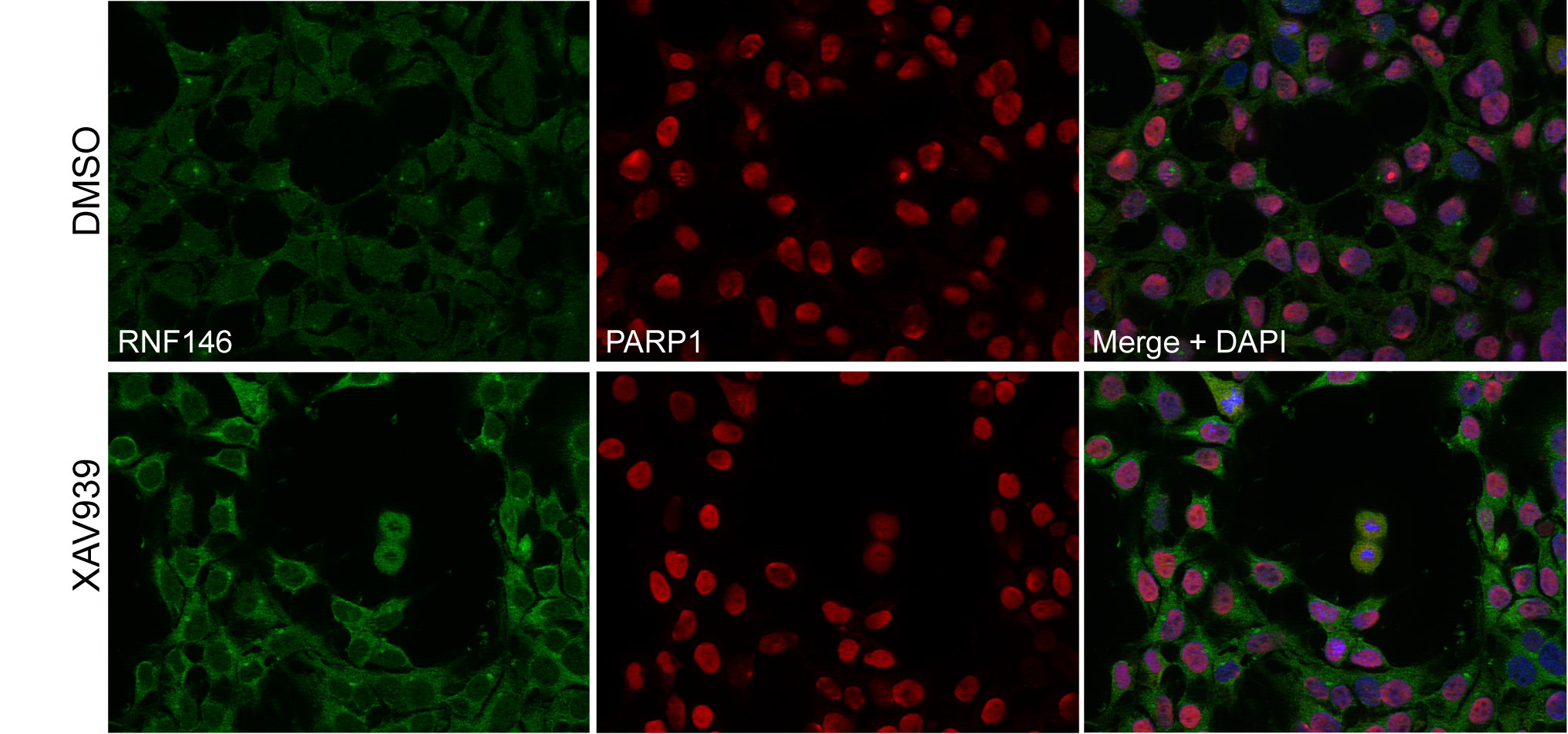

Supplement: Figure S4 — RNF146 RNAi does not affect PARP1 subcellular localization. HEK293 cells were treated with DMSO or XAV939 and immunostained for endogenous RNF146 (green) and PARP1 (red). DAPI counterstaining shows nuclear PARP1 in the merged image (magenta). (TIF) [file pone.0022595.s004.tif]
